# Colonic Hypermotility in a Rat Model of Irritable Bowel Syndrome Is Associated with Upregulation of TMEM16A in Myenteric Plexus

**DOI:** 10.1007/s10620-018-5261-7

**Published:** 2018-08-29

**Authors:** Meng-juan Lin, Bao-ping Yu

**Affiliations:** 1 Department of Gastroenterology, Renmin Hospital of Wuhan University, No. 238 Jiefang Rd, Wuhan, 430060 Hubei People’s Republic of China; 2 Key Laboratory of Hubei Province for Digestive System Diseases, Wuhan, People’s Republic of China

**Keywords:** Irritable bowel syndrome, Interstitial cells of Cajal, Myenteric plexus, Gastrointestinal motility

## Abstract

**Background:**

Irritable bowel syndrome (IBS) is a common disease with intestinal dysmotility, whose mechanism remains elusive. TMEM16A is a calcium-activated chloride channel (CaCC) involved in intestinal slow-wave generation.

**Aims:**

To investigate whether TMEM16A is involved in colonic dysmotility in IBS.

**Methods:**

A rat model of IBS was established by chronic water avoidance stress (WAS). Colonic pathological alterations were evaluated histologically, and intestinal motility was assessed by intestinal transit time (ITT) and fecal water content (FWC). Visceral sensitivity was determined by visceromotor response (VMR) to colorectal distension (CRD). TMEM16A expression was evaluated by RT-PCR, Western blot, and immunofluorescence. Colonic muscle strip contractility was measured by isometric transducers, and the effect of niflumic acid (NFA), a CaCC antagonist, on colonic motility was examined.

**Results:**

After 10 days of WAS exposure, ITT was decreased and FWC was elevated. Furthermore, VMR magnitude of WAS rats in response to CRD was significantly enhanced. Protein and mRNA levels of TMEM16A in colon were considerably increased after WAS. The percentage of TMEM16A-positive neurons in myenteric plexus (MP) of WAS rats was significantly higher than controls. Pharmacological blockade of TMEM16A activity by NFA considerably enhanced ITT, with concentration-dependent declines in FWC and VMR magnitude in NFA-treated rats. Further, spontaneous contraction of colonic strips of NFA-treated rats was significantly ameliorated in a concentration-dependent manner in vitro.

**Conclusions:**

Upregulation of TMEM16A in MP neurons may play an important role in chronic stress-induced colonic hypermotility, making CaCC-blocking drugs a putatively effective treatment method for colonic hypermotility in IBS.

## Introduction

Rhythmic contractions of gastrointestinal (GI) smooth muscle are the basis for GI motility, such as peristalsis and segmentation. Accumulating studies indicate that phasic contractions of GI tract are initiated and timed by slow wave generated by interstitial cells of Cajal (ICC) [1]. Slow waves, whose amplitude determines the opening of L-type Ca^2+^ channels in smooth muscle cells, are actively propagated within ICC networks and conducted to surrounding smooth muscle cells via gap junctions, accompanied by contractions [2]. It has been demonstrated that the amplitude and frequency of slow waves in the gut are regulated by excitatory and inhibitory enteric motor neurons of the enteric nervous system (ENS) [3, 4]. The ENS, which is regarded as a “brain-in-the-gut,” consists of two major populations of ganglia, the submucosal plexus (SMP) and the myenteric plexus (MP). The motor function for the specific digestive state of the gut is programmed by the MP [3, 4].

Irritable bowel syndrome (IBS), which is characterized by visceral hyperalgesia and intestinal dysmotility, is a common functional gastrointestinal disorder that affects up to 30% of the worldwide adult population [5]. However, the pathological mechanism of IBS is not well understood. There is accumulating evidence that dysfunction of ICC and ENS in the colon represents an important candidate mechanism responsible for intestinal dysmotility in IBS [6, 7]. Recent research has shown that slow waves in the GI tract are mediated by Ca^2+^-activated Cl^−^ channels (CaCC), most likely encoded by *TMEM16A* (also known as *ANO1* or *DOG1*) in ICC [8–10]. TMEM16A is involved in the generation of a Ca^2+^-activated Cl^−^ inward current in ICC [11]; it was first found in gastrointestinal stromal tumors (GIST) and has been recently reported as a sensitive and specific marker for GIST [12]. The CaCC channel blocking drugs niflumic acid (NFA) and 4,4′-diisothiocyano-2,2′-stillbene-disulfonic acid (DIDS) specifically block slow waves in intact muscle of small intestine and stomach in mouse, primate, and human [8]. Importantly, slow waves fail to develop in TMEM16A knockout mice [9]. A more recent study indicates that conditional genetic deletion of TMEM16A also impairs Ca^2+^ transients in ICC of adult mouse small intestine [13].

However, it remains unclear whether TMEM16A mediates stress-induced GI dysmotility. Herein, the present study was designed to explore alterations of expression and distribution of TMEM16A in the colon and to determine the role of TMEM16A in intestinal dysmotility in a rat model of IBS induced by chronic stress.

## Materials and Methods

### Animals

Male Sprague–Dawley rats (weight 180–230 g) were obtained from Hunan SJA Laboratory Animal Co., Ltd. The animals were habituated to standard laboratory conditions (22 ± 2 °C with a 12 h light/dark cycle and a relative humidity of 40–60%) and provided with food and water ad libitum. All experiments were approved by the Institutional Animal Care and Use Committee of Wuhan University and were conducted in accordance with the Declaration of the National Institutes of Health Guide for Care and Use of Laboratory Animals and the People’s Republic of China animal welfare legislations in order to minimize the number of experimental animals and their suffering.

The rats were randomly divided into five groups (*n* = 12/group): control group, water avoidance stress (WAS) group, NFA-low (NFA-L) group (0.1 mg/kg), NFA-medium (NFA-M) group (0.4 mg/kg), and NFA-high (NFA-H) group (1 mg/kg). The WAS procedure was performed to induce IBS as described previously with minor modifications [14]. Briefly, rats were placed on a platform (10 × 8 × 8 cm; length × width × height) in the center of a water-filled (25 °C) tank (45 × 25 × 35 cm; length × width × height) for 1 h daily for ten consecutive days. The water level in the tank was kept at 1 cm below the platform. The animals from the NFA-treated group were administered an intraperitoneal injection of NFA (Aladdin, Shanghai, China) in different doses, in saline, 1 h before WAS since the fourth day of WAS for 7 days. After 48 h, colonic motility and visceral sensitivity were determined. Afterward, the rats were subjected to laparotomy and distal colon resection. Hematoxylin–eosin staining was performed for colon specimens.

### Intestinal Transit Time (ITT)

The animals were orally gavaged with activated carbon in double-distilled water. Closely observation of the stool was then conducted. ITT was the duration from gavage to the time when the first black fecal pellet was output.

### Fecal Water Content (FWC)

FWC was used to estimate colonic motility as a validated index. The animals were placed in metabolic cages for 24 h with free access to rodent chow and water. The stool was weighed (*m*_0_) after collection, and then the stool was weighed again (*m*_1_) after the stool was dried in the oven. FWC was calculated as (*m*_0_ − *m*_1_)/*m*_0_.

### Electromyogram (EMG) Measurements

To evaluate the visceral hyperalgesia, we recorded the EMG signal of abdominal oblique musculature. For EMG measurements, animals were initially anesthetized with isoflurane inhalation, keeping a mild and stable anesthesia throughout the experiment. After anesthesia, the rat was fixed in a supine position. A pair of electrodes was implanted into the external oblique muscle of the rats. The electrodes were connected to a Bio Amp (AD instruments, Bella Vista, Australia), which was connected to a Power Lab (AD instruments, Bella Vista, Australia) as an EMG acquisition system. Colorectal distension (CRD) was then conducted after 20 min of adaptation. Each recording progression consisted of a 5-min predistention baseline activity measurements, a 20-s CRD-evoked response (20, 40, 60, and 80 mmHg), and a 3-min postdistention activity measurement, followed by a 3-min rest between two CRD episodes. The EMG signals expressed by visceromotor response (VMR), the area under the curve (AUC) in response to the CRD stimuli, were collected and analyzed using Lab Chart 7 software (AD instruments, Bella Vista, Australia). The analytic period was 40 s (20 s during and 20 s after each CRD). The net value for each CRD was calculated by subtracting the AUC of the baseline (40 s interval) before each CRD [15].

### Western Blot

Total proteins were extracted using RIPA lysis buffer (Beyotime, Shanghai, China) and subsequently subjected to centrifugation at 12,000 rpm, 4 °C for 30 min. Supernatants were then collected and protein concentrations were determined using the BCA protein assay kit (Beyotime, Shanghai, China). Samples were mixed with 5 × loading buffer and heated at 100 °C for 5 min to denature the proteins. Thirty micrograms of total proteins was loaded on 10% SDS polyacrylamide gels and electrophoresed. The separated proteins were transferred to PVDF membranes (Millipore, Darmstadt, Germany), and the membranes were incubated in 5% skimmed milk at room temperature for 2 h to block nonspecific binding. The blots were then incubated overnight at 4 °C with the primary antibody against TMEM16A (Santa Cruz, California, USA) and β-actin (Beyotime, Shanghai, China). After washing three times with TBST for 10 min, the corresponding secondary antibody conjugated to horseradish peroxidase (Boster, Wuhan, China) was applied for 1 h at room temperature, followed by three washes of TBST for 10 min. Specific protein bands were visualized using the ECL kit (Thermo, Massachusetts, USA) and an X-ray film (Kodak, Xiamen, China). The optical density of the bands was analyzed using Band Scan 5.0 software (Alpha Innotech Corp., California, USA).

### Quantitative RT-PCR

Total RNA was extracted from the colon that was frozen in liquid nitrogen with TRIzol reagent (Invitrogen, Carlsbad, USA), following the manufacturer’s instructions. Next, 1.5 µg of total RNA from each sample was used for cDNA synthesis in a total volume of 20 µl, with oligo (dT)18 and HiScript Reverse Transcriptase (VAZYME, Nanjing, China) included in the reverse transcription system. Quantitative RT-PCR was performed in 20 µl wells with SYBR green PCR master mix (VAZYME, Nanjing, China) on the ViiA 7 real-time PCR system (ABI, Carlsbad, USA). After incubation at 95 °C for 10 min as the initiation of thermal cycling, 40 cycles of 95 °C for 30 s and 60 °C for 30 s were performed. Each reaction was performed in triplicate. GAPDH was used as a loading control to normalize each sample. The PCR primers used were as follows: TMEM16A forward: 5′-TGGGCTACGAGGTTCAGATC-3′, reverse: 5′-TGGCTGATGTCTTTGGGGAT-3′; GAPDH forward: 5′-ACAGCAACAGGGTGGTGGAC-3′, reverse: 5′-TTTGAGGGTGCAGCGAACTT-3′. Specificity of the PCR products was monitored by melting curve analysis. The relative expression of TMEM16A mRNA was quantified by the 2^−∆∆Ct^ method.

### Immunofluorescence

Immunofluorescence staining for TMEM16A was performed both on the whole-mount flat preparations and longitudinal sections of the colon. Immunofluorescence staining for TMEM16A on whole-mount flat preparations was performed as follows. Colonic specimens were fixed in 4% paraformaldehyde solution at 4 °C for 6-8 h. Subsequently, the mucosa was removed with sharp forceps under a stereoscopic microscope. The circular muscle was then stripped off carefully at certain intervals. Whole-mount preparations were then blocked with 10% goat serum containing 0.3% Triton X-100 for 1 h at room temperature. Next, the samples were incubated overnight at 4 °C with rabbit anti-rat polyclonal TMEM16A antibody (Santa Cruz, California, USA). After washing in PBST, the pinned tissues were incubated overnight at 4 °C with mouse anti-rat monoclonal PGP9.5 antibody (Abcam, Cambridge, England). After washing thrice with PBST for 5 min, the tissues were incubated with TRITC-conjugated goat anti-rabbit secondary antibody (Boster, Wuhan, China) and FITC-conjugated rabbit anti-mouse secondary antibody (Boster, Wuhan, China) for 1 h at room temperature. The stained samples were imaged using an Olympus BX53 microscope (Olympus, Tokyo, Japan) after they were mounted on a slide with a coverslip and sealed with glycerol. The results were analyzed using Image Pro Plus software version 6.0. The TMEM16A-immunoreactive (IR) neurons were quantified as a relative percentage considering the total number of PGP9.5-IR neurons. Immunofluorescence of colonic longitudinal sections was conducted in a similar manner using the above procedure.

### Contractility of Colonic Muscle Strips

Full-thickness strips of distal colon (measuring 3 × 10 mm) were mounted vertically in a 10-ml organ bath filled with Tyrode’s solution maintained at 37 °C and constantly bubbled with O_2_. The strips were placed under an initial resting tension equivalent to a 1.0 g load and allowed to equilibrate for 30 min, with solution changing every 20 min. Isometric contractions were measured using a force displacement transducer. The contraction curves were recorded and measured by RM6240 multichannel physiological signal acquisition and processing system. Changes in average magnitude, frequency, and area under the contraction curve (AUC) were calculated to evaluate the spontaneous contraction of strips.

### Statistical Analysis

Statistical analyses were performed using SPSS version 21.0 (IBM Co, Chicago, USA). Continuous variables were presented as mean ± standard deviation (SD) and compared using *t test* and variance analysis. Differences among different groups were analyzed by two-way repeated-measures analysis of variance (ANOVA) with distention pressure as the repeated measure. S–N–K post hoc test was used where appropriate. A two-sided *P* value < 0.05 was regarded as statistically significant.

## Results

### Evaluation of the Animal Model

**Fig. 1:**
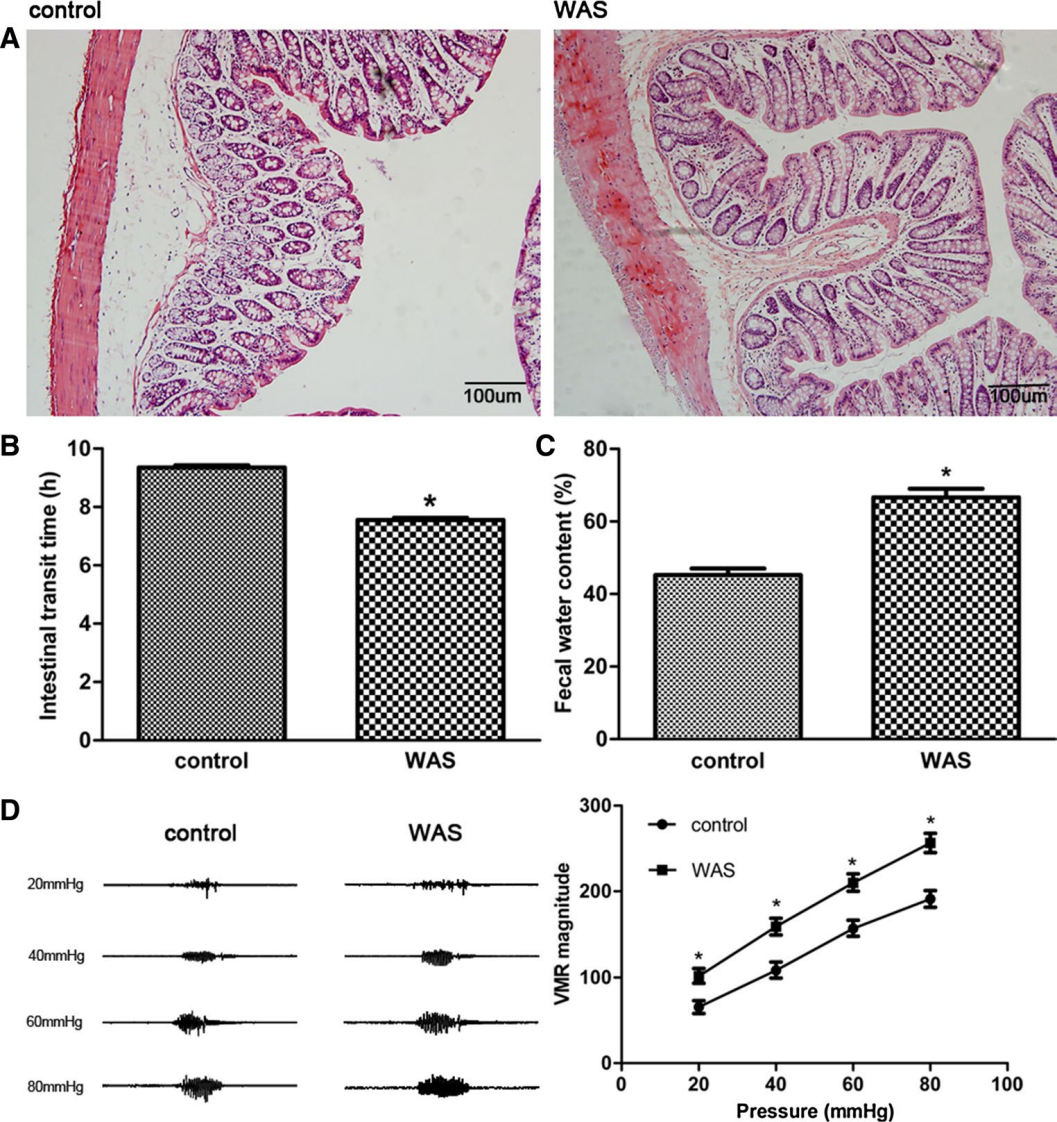
Evaluation of the animal model. **a** Hematoxylin–eosin staining (×200) shows colon specimens of control (left) and WAS (right) groups are intact, without congestion and obvious infiltration of inflammatory cells. **b** ITT of WAS rats is significantly shorter than that of control rats. **c** FWC of the WAS group is enhanced compared to controls. **d** VMR curve (left) and summary data (right) for raw VMR responses to CRD at pressures of 20, 40, 60, and 80 mmHg in control and WAS-exposed rats. VMR amplitude of the WAS group is markedly higher than that of the control group. *P < 0.05 compared to controls (both groups *n* = 6)

No rats died during the experiment. Hematoxylin–eosin staining revealed that colon specimens of the control and WAS groups were intact, simultaneously without congestion and obvious infiltration of inflammatory cells (Fig. 1a). However, ITT of WAS rats (7.72 ± 0.32 h) was significantly shorter than that of the control group (9.43 ± 0.21 h) (Fig. 1b). Moreover, FWC of the WAS group (66.63%) was enhanced compared to the control group (45.65%) (Fig. 1c). Furthermore, we detected a significantly higher VMR amplitude of WAS-exposed rats than that found in control rats (20 mmHg: control 65.3 ± 7.8 μv s vs. WAS 101.6 ± 8.6 μv s, *P* < 0.05; 40 mmHg: control 108.4 ± 9.5 μv s vs. WAS 159.1 ± 9.4 μv s, *P* < 0.05; 60 mmHg: control 157.1 ± 9.3 μv s vs. WAS 210.4 ± 10.3 μv s, *P* < 0.05; 80 mmHg: control 191.4 ± 9.8 μv s vs. WAS 256.8 ± 11.4 μv s, *P* < 0.05; control and WAS groups, both *n* = 6) (Fig. 1d).

These results suggest that an animal model of chronic stress-induced colonic hypermotility, exhibiting the hallmark of IBS, was successfully established after 10 days of WAS exposure.

### Increased TMEM16A Expression in Colon of WAS Rats

**Fig. 2:**
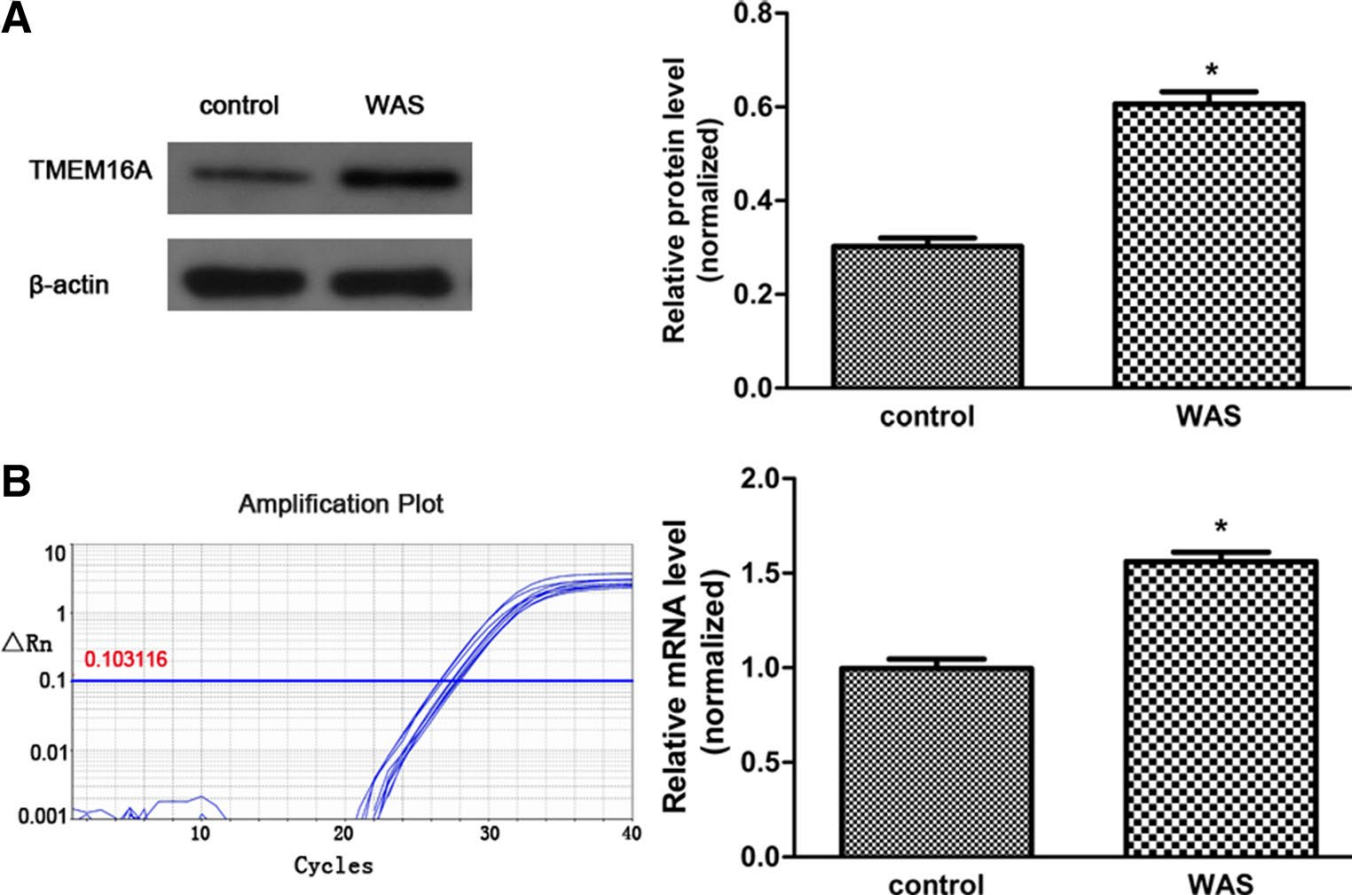
Protein and mRNA levels of TMEM16A in the colon. **a** Western blot analysis reveals increased TMEM16A protein expression in WAS rats compared to control rats. **b** Quantitative RT-PCR analysis shows that TMEM16A mRNA levels in WAS rats are increased compared to those found in controls. *P < 0.05 compared to controls (all groups *n* = 6)

Western blot analysis revealed that TMEM16A protein level in the colon was markedly increased after 10 days of WAS treatment (Fig. 2a), with ratios of TMEM16A to β-actin in control and WAS groups of 0.3017 ± 0.03216 and 0.6067 ± 0.04361 (*P* < 0.05), respectively. Quantitative RT-PCR analysis further confirmed upregulation of TMEM16A in the colon under chronic stress at the transcriptional level (Fig. 2b). We found that the mean 2^−ΔΔCt^ values of TMEM16A mRNA levels in control and WAS groups were 1.0000 ± 0.0000 and 1.569 ± 0.0376 (*P* < 0.05), respectively.

These results indicate that TMEM16A expression in the colon was considerably upregulated after ten consecutive days of WAS exposure.

### Immunofluorescence Staining of TMEM16A in the Colon

**Fig. 3:**
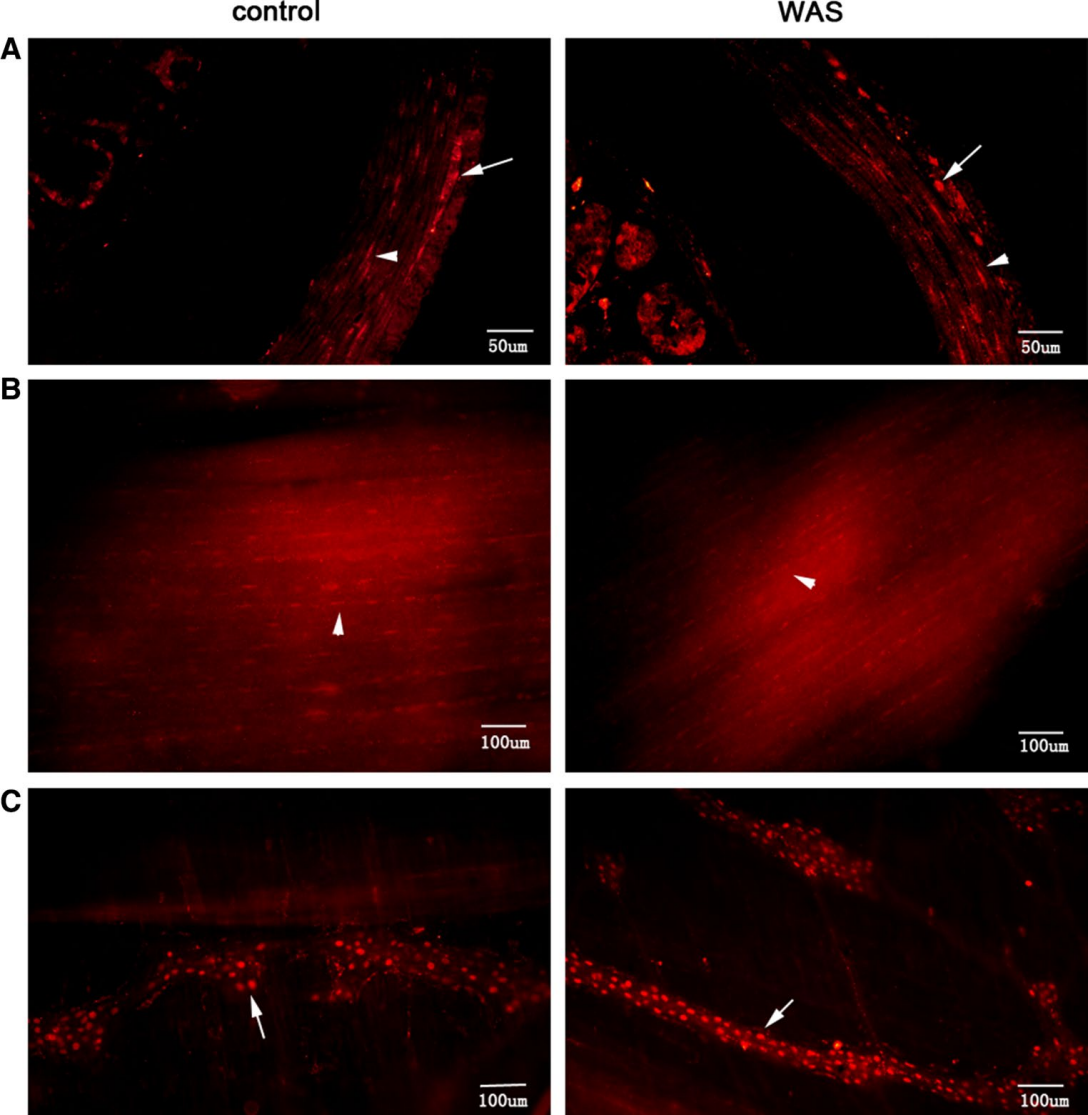
Immunostaining of TMEM163A in the colon. **a** Immunofluorescence staining for TMEM16A in longitudinal colonic paraffin sections from control (left) and WAS-exposed (right) rats (×400) reveals that the number of TMEM16A-IR cells in MP of WAS rats is significantly increased compared to control rats. **b** Immunofluorescence of whole-mount circular muscle (×200) reveals TMEM16A-positive cells are mainly fusiform in shape. Immunofluorescence analysis confirms an equivalent density of TMEM16A-positive labeling between WAS (right) and control (left) groups. **c** Immunofluorescence for TMEM16A on whole-mount preparations of colonic MP (×200) illustrates TMEM16A is robustly expressed in colonic MP. TMEM16A-IR cells are predominantly round or oval in shape. White arrowheads indicate TMEM16A-positive cells that are mainly fusiform in shape and found within circular muscle, exhibiting the morphological characteristics of ICC. White arrows indicate TMEM16A-positive cells that are predominantly round in shape and located between circular and longitudinal muscles, showing the hallmark of MP neurons (both groups *n* = 6)

From immunofluorescence staining of longitudinal paraffin sections, we clearly observed that two populations of TMEM16A-positive cells were present in the muscular layer (Fig. 3a). One population was predominantly fusiform in shape and was found within circular muscle, parallel to smooth muscle cells and exhibiting the morphological characteristics of ICC, while the other population was mainly round in shape located between circular and longitudinal muscles, showing the hallmark of MP neurons. Immunofluorescence analysis revealed that the number of TMEM16A-IR cells in the MP of WAS rats was significantly increased compared to that found in control rats (11.47 ± 2.71 vs. 7.16 ± 2.54, respectively, *P* < 0.05); however, the number of TMEM16A-IR cells in the circular muscle of WAS rats was comparable to that of controls (10.52 ± 3.11 vs. 9.76 ± 3.62, respectively, *P* > 0.05).

Furthermore, immunofluorescence of whole-mount circular muscle revealed that TMEM16A-positive cells, mainly intramuscular ICC, were largely fusiform in shape with two prolongations connecting each other in a head-to-tail sequence and parallel to smooth muscle cells (Fig. 3b). Immunofluorescence analysis confirmed an equivalent density of TMEM16A labeling between WAS and control rats (*P* > 0.05).

**Fig. 4:**
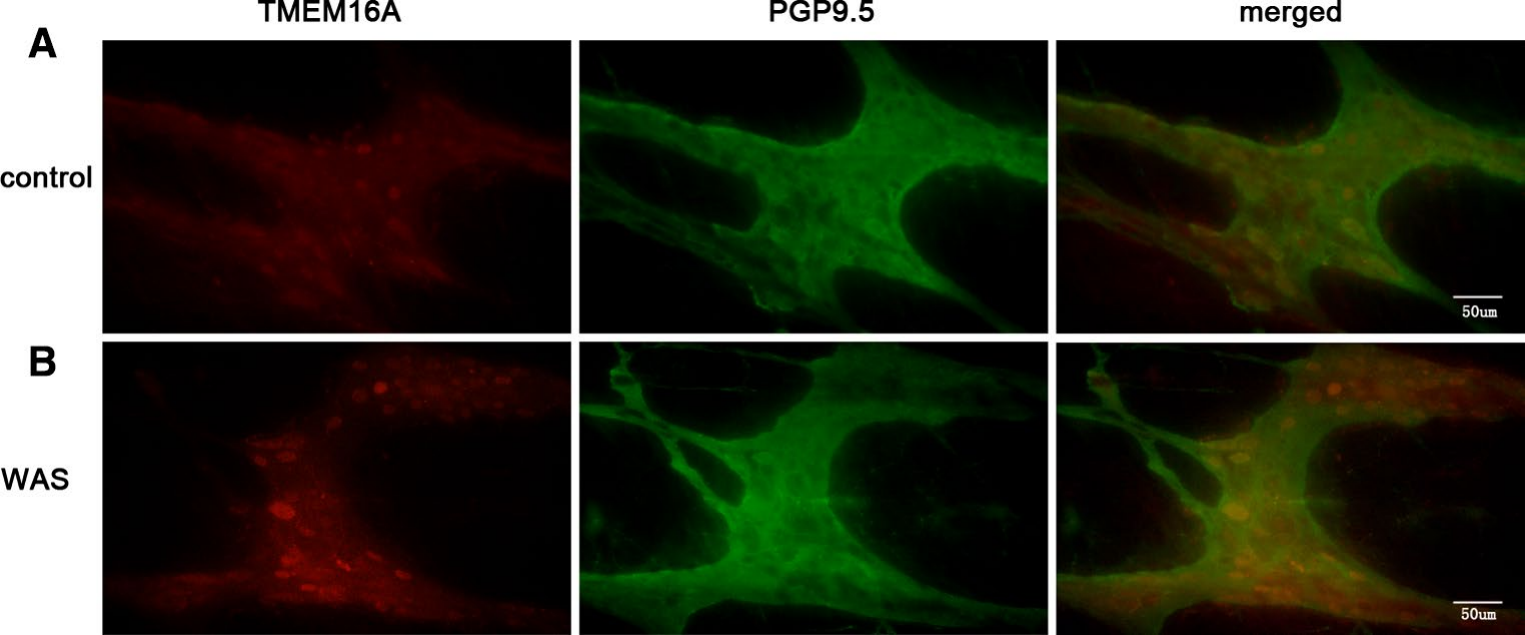
Distribution of TMEM16A-IR cells in the MP of colon. Immunofluorescence of CHT1, PGP9.5, and merged pictures on whole-mount preparations of colonic MP from control (**a**) and WAS (**b**) rats (×400) are displayed. The neuronal network-like structure of the MP is clearly observed. The percentage of TMEM16A-positive neurons in the MP of WAS-exposed rats is significantly higher than that found in MP of control rats (both groups *n* = 6)

Next, we examined the distribution of TMEM16A-positive cells in the MP by stripping the circular muscle off whole-mount colonic specimens. We detected robust expression of TMEM16A in the colonic MP, and TMEM16A-IR cells were predominantly round or oval in shape (Fig. 3c). Furthermore, the distribution of TMEM16A-IR neurons in the MP of the distal colon was examined by identifying MP neurons with an anti-PGP9.5 antibody. The neuronal network-like structure of MP was clearly observed on the colonic MP whole mounts after immunostaining (Fig. 4). We observed that all TMEM16A-IR cells in MP were co-labeled with PGP 9.5. Compared to control rats (56.76 ± 3.52%), there was a significantly increased percentage of TMEM16A-IR neurons in MP of WAS-exposed rats (85.47 ± 5.71%) (*P* < 0.05).

These findings show that the enhanced expression of TMEM16A observed in WAS rats was restricted to MP neurons of the ENS rather than ICC.

### NFA-Alleviated Colonic Hypermotility in a Concentration-Dependent Manner In Vivo

**Fig. 5:**
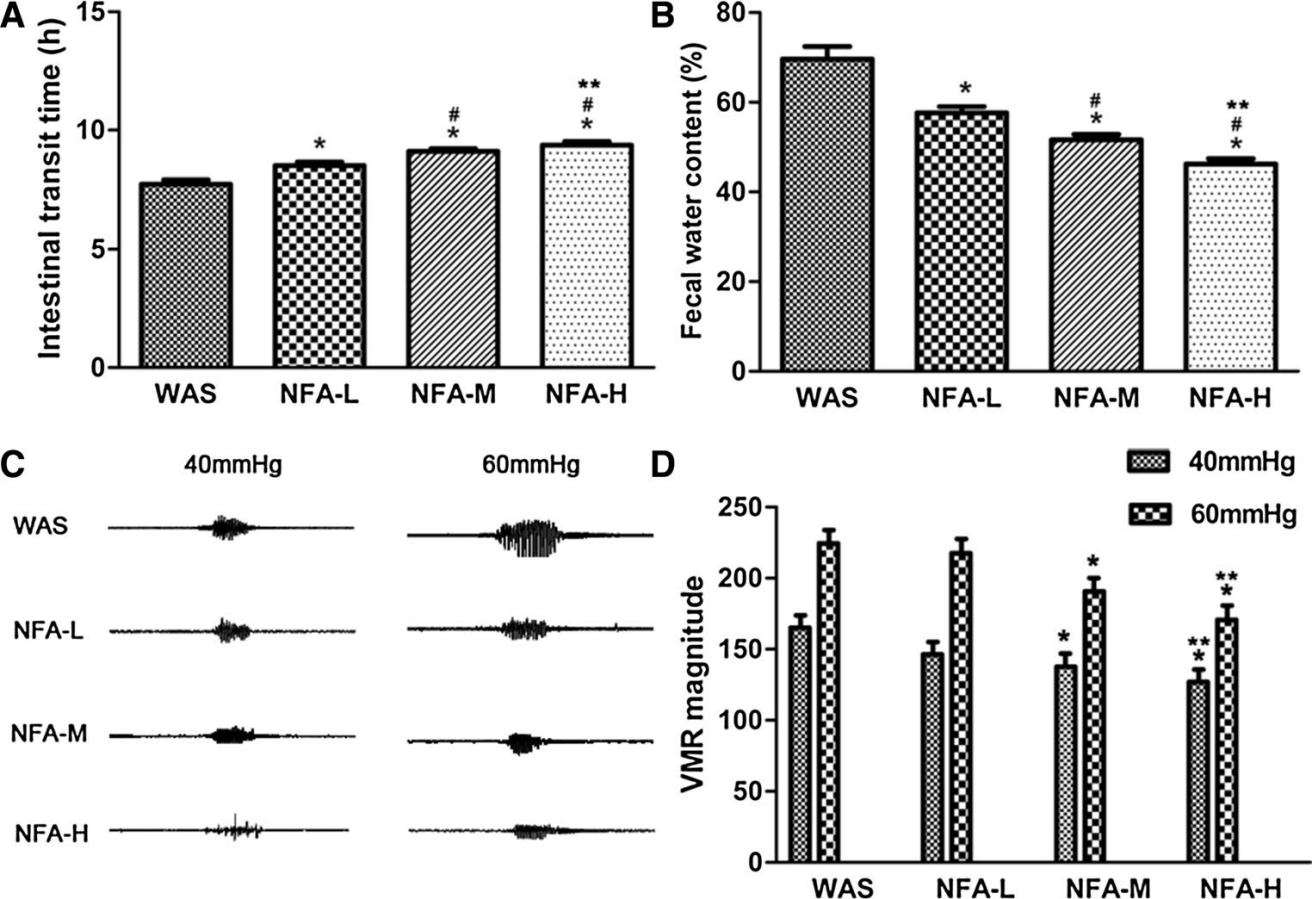
Influence of NFA on intestinal motility in vivo. **a** ITT of NFA-treated rats are significantly prolonged compared to WAS rats in a concentration-dependent manner. **b** Compared to WAS rats, FWC of NFA-treated rats is attenuated with increasing concentrations of NFA. **c** Medium and high doses of NFA reverse the VMR amplitude observed in WAS rats. *P < 0.05, compared to WAS-exposed rats; ^#^*P* < 0.05 compared to NFA-L group; **P < 0.05 compared to NFA-M group (all groups *n* = 6)

The selective CaCC antagonist NFA was administered to explore the role of TMEM16A in stress-induced intestinal hypermotility. We found that ITT of WAS-exposed rats was significantly prolonged after treatment with NFA (7.72 ± 0.32 h in WAS group vs. 8.53 ± 0.27 h in NFA-L group, 9.11 ± 0.23 h in NFA-M group, and 9.37 ± 0.28 h in NFA-H group, *P* < 0.05) (Fig. 5a). Furthermore, compared to WAS rats, FWC of NFA-treated rats was attenuated with increasing NFA concentration (68.31% in WAS group vs. 57.71% in NFA-L group, 51.63% in NFA-M group, and 47.51% in NFA-H group, *P* < 0.05) (Fig. 5b). In addition, medium and high doses of NFA reversed VMR amplitude (40 mmHg: WAS 165.1 ± 8.7 μv s vs. NFA-L 146.3 ± 9.1 μv s, NFA-M 137.8 ± 9.2 μv s, and NFA-H 126.9 ± 9.0 μv s, *P* < 0.05; 60 mmHg: WAS 224.6 ± 8.3 μv s vs. NFA-L 217.6 ± 10.1 μv s, NFA-M 190.8 ± 9.4 μv s, and NFA-H 170.9 ± 9.8 μv s, *P* < 0.05) (Fig. 5c, d).

These results suggest that NFA, a blocker of TMEM16A, alleviated colonic hypermotility in WAS rats in a concentration-dependent manner in vivo.

### NFA-Attenuated Spontaneous Contraction of Colonic Strips in a Concentration-Dependent Manner In Vitro

**Fig. 6:**
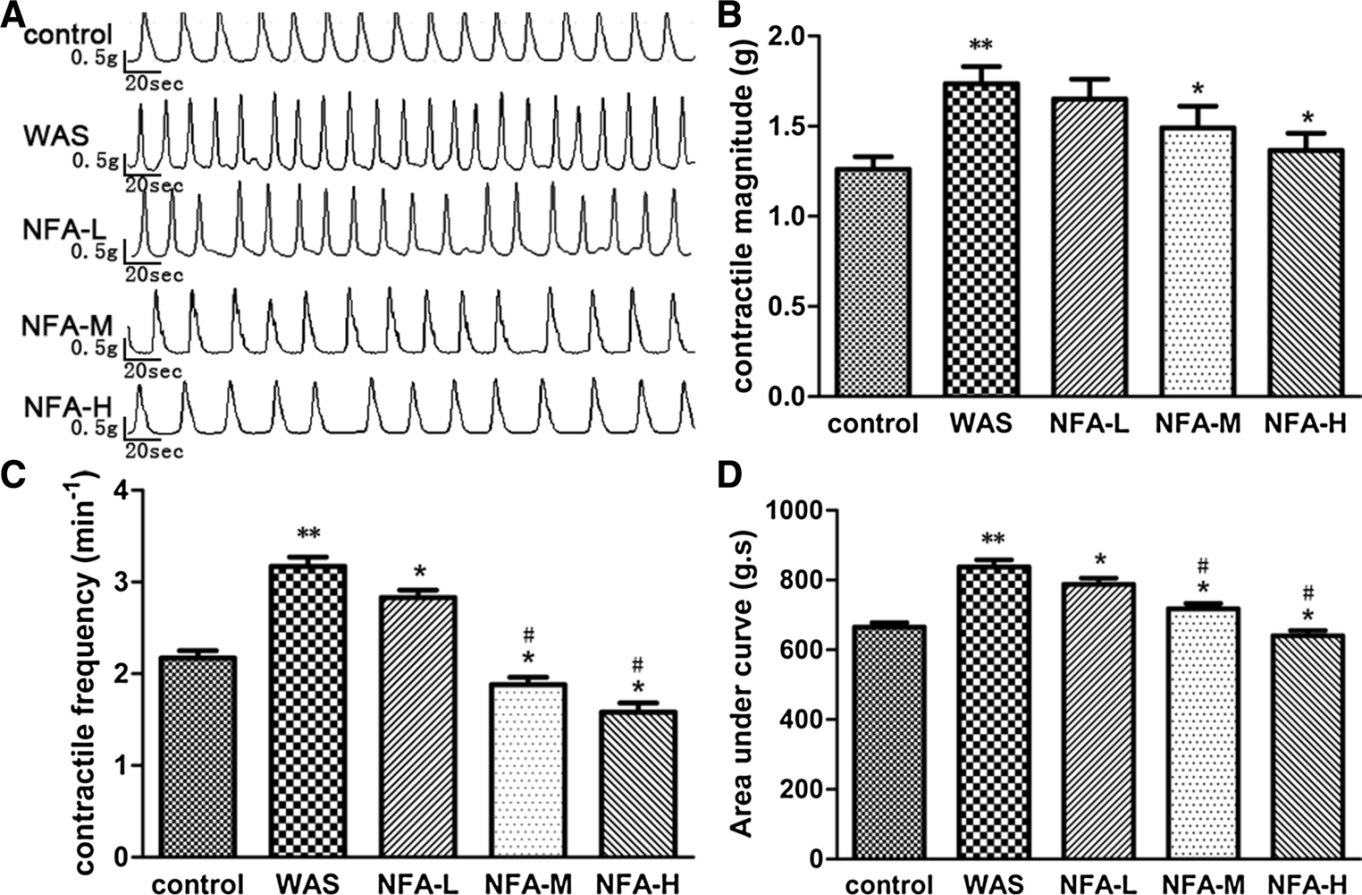
Effect of NFA on spontaneous contraction of colonic strips. **a** A representative image showing spontaneous contraction of colonic strips in vitro. **b** The average magnitude of WAS rats is significantly increased compared to that found in control rats, while the average magnitude of colonic strips of the NFA-M and NFA-H groups is declined compared to WAS-exposed rats, exhibiting a concentration-dependent decline with increasing NFA concentration. **b** Frequency and **c** AUC of the NFA-treated group are significantly lower than those of WAS rats. However, higher doses (NFA-H and NFA-M) exert greater effects on relief of colonic hypermotility than a lower dose (NFA-L). *P < 0.05 compared to WAS-exposed group; **P < 0.05 compared to controls; ^#^*P* < 0.05 compared to NFA-L group (all groups *n* = 6)

In the current study, spontaneous contractility of colonic strips was evaluated using changes in the average magnitude, frequency, and area under the contraction curve (Fig. 6). Notably, the average magnitude of rats from the WAS group was significantly higher than that found in control rats (1.734 ± 0.17 g vs. 1.263 ± 0.12 g, respectively, *P* < 0.05). Conversely, the average magnitude of colonic strips of rats from the NFA-M or NFA-H group was decreased compared to that of WAS-exposed rats, exhibiting a stepwise decline with increasing concentrations of NFA (1.734 ± 0.17 g in WAS group vs. 1.489 ± 0.21 g in NFA-M and 1.367 ± 0.16 g in NFA-H groups, *P* < 0.05). However, a low dose of NFA (1.652 ± 0.19 g in NFA-L group) had no effect on reversing the increased magnitude observed in WAS rats. In addition, the frequency (2.169 ± 0.15 min^−1^ in control group; 3.171 ± 0.17 min^−1^ in WAS group vs. 2.832 ± 0.13 min^−1^ in NFA-L group, 1.896 ± 0.14 min^−1^ in NFA-M group, and 1.586 ± 0.16 min^−1^ in NFA-H group, *P* < 0.05) and AUC (664.72 ± 23.54 g·s in control group; 837.17 ± 36.84 g·s in WAS group vs. 786.83 ± 34.31 g·s in NFA-L group, 716.67 ± 28.90 g·s in NFA-M group, and 639.28 ± 26.78 g.s in NFA-H group, *P* < 0.05) of NFA-treated groups were significantly lower than those of the WAS-treated group. However, medium and high doses of NFA have a greater effect on relief of colonic hypermotility than low dose of NFA (*P* < 0.05).

These findings indicate that NFA relieved chronic stress-induced spontaneous contractility of colonic strips in a concentration-dependent manner in vitro.

## Discussion

Findings from recent studies that TMEM16A functions as a classical Ca^2+^-activated Cl^−^ channel have ignited significant interest in this new family of ion channels [16]. TMEM16A is widely expressed in many organs including lung, salivary gland, intestine, kidney and other tissues, as well as arterial smooth muscle, intestinal pacemaker cells, and sensory neurons, in which TMEM16A facilitates epithelial fluid secretion, smooth muscle contraction, and neurosensory signaling [9, 17–19]. TMEM16A is robustly expressed in GI muscular layer, specifically in ICC of murine, non-human primate, and human [9, 20]. Recent studies have demonstrated that TMEM16A, non-selective cation channels (e.g., transient receptor potential channels) and sodium channels are the three most important ion channels in generation of the ICC slow wave in GI tract [21–23]. In the present study, we investigated the role of TMEM16A in colonic motility dysfunction in a rat model of IBS.

Presently, the detailed mechanism of IBS remains elusive. Therefore, animal models of IBS are pivotal in clarifying its pathogenesis. To date, repeated WAS has frequently been utilized to establish animal models of stress-induced IBS with visceral hypersensitivity, motility impairment, anxiety, and colonic immune activity [24]. Currently, the WAS-based model optimum for studies on IBS is a brain-gut interaction model that mimics some clinical and pathophysiological characteristics of IBS-diarrhea [25]. In the present study, enhanced gut motility was observed after 10 days of WAS exposure, indicating that an animal model of stress-induced dysmotility in gut was successfully established in our study. A more recent study by Reed et al. [26] suggested that myenteric neurons play a key role in gut motor dysfunction of WAS-exposed rats. We discovered that molecular alterations of myenteric neurons in colonic MP could give rise to the gut dysmotility in IBS induced by WAS, which importantly adds to the currently limited published literature available on this condition.

Recent evidence indicates that expression of TMEM16A could be regulated by metabolic disease [27], dysplasia [28], and carcinoma [11] in specific parts of the GI tract, such as gastric, small intestine, and colon. A previous study identified important changes in expression and splicing of TMEM16A in patients with diabetic gastroparesis [27]. Further, TMEM16A-positive ICC in tissues of patients with slow transit constipation displayed a significant decline compared to that found in healthy individuals [28]. However, it remains elusive whether stress modulates TMEM16A expression in the GI tract. In our colonic hypermotility model, we detected enhanced expression of TMEM16A in both protein and mRNA levels in colon after ten consecutive days of WAS exposure, indicating TMEM16A expression could be directly regulated by chronic stress. Based on these findings, we posit that TMEM16A is involved in the pathological course of motility disorder in GI tract.

Accumulating studies have confirmed that TMEM16A is exclusively expressed in ICC of the muscular layer of the GI tract [29] and is a highly specific biomarker of ICC, exhibiting more selective labeling of ICC than Kit antibodies [20]. However, in the present study, we clearly observed two distinct populations of TMEM16A-positive cells in the muscular layer of colon, namely ICC of the intramuscular region and neurons of the MP. To date, this is the first study to report the presence of TMEM16A-positive neurons in the ENS. Furthermore, we detected an increased density of TMEM16A-positive MP neurons in WAS-exposed rats. Our data indicate that chronic stress-driven changes in gut motility suggest direct modulation of ENS, providing important evidence in support of this mechanism in mediating stress-driven changes on gut-brain signaling. Nitrergic and cholinergic neurons of the ENS are the principal inhibitory and excitatory musculomotor neurons of gut, respectively [30]. Certain studies have suggested that Ca^2+^-activated Cl^−^ conductance generated by TMEM16A in ICC, which is likely activated by acetylcholine (ACh), plays a pivotal role in the function of gut excitatory motor neurons [31–33]. According to our data, TMEM16A is robustly expressed in neurons of the colonic MP and displays an increasing trend in the ENS of WAS rats. One reasonable explanation for the colonic hypermotility in IBS may be that enhanced TMEM16A expression in the MP neurons regulates excitability of excitatory and inhibitory motor neurons. The subsequent release of neurotransmitters, such as ACh and nitric oxide, induces a more extensive depolarization in gut smooth muscle via activation of TMEM16A in ICC.

The inhibitory effect of TMEM16A blockade in both ICC and GI smooth muscle has been previously investigated [8]. It is suggested that NFA causes concentration-dependent reductions in the amplitude of slow-wave inward current, resulting in reduced frequency, upstroke velocity, and duration of slow waves in ICC under current clamp [8]. In addition, NFA blocks slow waves in intact muscle of mouse and primate, as well as human small intestine and stomach [8]. The findings from this report suggest that NFA could weaken GI motility by impairing the slow wave in ICC and whole muscle, implying an important role of TMEM16A in normal GI motility. Here, we demonstrate that NFA alleviates colonic hypermotility in WAS-exposed rats both in vivo and in vitro. Based on our findings, we conclude that enhanced TMEM16A expression in colon may play an important role in intestinal hypermotility, and therefore, downregulation of TMEM16A or TMEM16A blockade may be used to treat disorders with GI hypermotility.

A limitation of the present study is that electrophysiological alterations of TMEM16A were not examined. Although our data imply a role for TMEM16A in chronic stress-induced colonic dysmotility, it is not known whether altered Cl^−^ homeostasis also contributes to muscle contraction in common chronic functional bowel disorders such as IBS. Therefore, further study focusing on electrophysiological alterations of TMEM16A in WAS rats is warranted.

In conclusion, our findings demonstrate that chronic stress-induced colonic motility dysfunction is associated with enhanced expression of TMEM16A in colonic muscular layer, especially in MP neurons. Further, these findings may contribute to the identification of new mechanisms underlying functional colonic hypersensitivity associated with enhanced stress responsiveness and may pave the way for novel treatments of IBS and related disorders.
